# Evaluation of Factors Influencing the Groundwater Chemistry in a Small Tropical Island of Malaysia

**DOI:** 10.3390/ijerph10051861

**Published:** 2013-05-06

**Authors:** Nura Umar Kura, Mohammad Firuz Ramli, Wan Nur Azmin Sulaiman, Shaharin Ibrahim, Ahmad Zaharin Aris, Adamu Mustapha

**Affiliations:** 1 Faculty of Environmental Studies, Universiti Putra Malaysia, 43400 UPM Serdang, Selangor, Malaysia; E-Mails: nuraumar@gmail.com (N.U.K.); wannor@env.upm.edu.my (W.N.A.S.); shaharin@env.upm.edu.my (S.I.); 2 Environmental Forensics Research Centre, Faculty of Environmental Studies, Universiti Putra Malaysia, 43400 UPM Serdang, Selangor, Malaysia; E-Mails: zaharin@env.upm.edu.my (A.Z.A.); amustapha494@gmail.com (A.M.)

**Keywords:** groundwater, hydrochemistry, island, multivariate statistics, pollution, seawater intrusion

## Abstract

Groun in a very complex way. In this work, multivariate statistical analysis was used to evaluate the factors controlling the groundwater chemistry of Kapas Island (Malaysia). Principal component analysis (P dwater chemistry of small tropical islands is influenced by many factors, such as recharge, weathering and seawater intrusion, among others, which interact with each other CA) was applied to 17 hydrochemical parameters from 108 groundwater samples obtained from 18 sampling sites. PCA extracted four PCs, namely seawater intrusion, redox reaction, anthropogenic pollution and weather factors, which collectively were responsible for more than 87% of the total variance of the island’s hydrochemistry. The cluster analysis indicated that three factors (weather, redox reaction and seawater intrusion) controlled the hydrochemistry of the area, and the variables were allocated to three groups based on similarity. A Piper diagram classified the island’s water types into Ca-HCO_3_ water type, Na-HCO_3_ water type, Na-SO_4_-Cl water type and Na-Cl water type, indicating recharge, mixed, weathering and leached from sewage and seawater intrusion, respectively. This work will provide policy makers and land managers with knowledge of the precise water quality problems affecting the island and can also serve as a guide for hydrochemistry assessments of other islands that share similar characteristics with the island in question.

## 1. Introduction

Groundwater is becoming the main source of freshwater for domestic, agricultural and other human activities in many places, particularly in coastal areas [1] where rapid population growth and intensive economic activities are pushing the demand for fresh water to its limits [2]. This is even more severe in small tropical islands such as Manukan Island (Malaysia) where groundwater has no substitute in providing fresh water [3], despite the high amounts of annual rainfall (more than 2,000 mm) such islands receive, especially during monsoon season [4]. Most of this rainwater is being lost to the sea through runoff before recharging the aquifer, mainly due to the topographical and hydrogeological characteristics of the islands. Moreover, exploitation of groundwater resources is escalating in attempts to meet the increasing demands, particularly of the fast growing tourism industry [5]. This can lead to the deterioration of groundwater by seawater intrusion, which has been identified as one of the major problems affecting groundwater [4]. In addition, complex interactions of multiple factors such as geology, mineral composition of an aquifer, weathering, water-rock interactions, topography, tidal effects, climate and anthropogenic activities are important determinants of groundwater quality [6,7,8]. Therefore, effective pollution control and sustainable water resources management are necessary to tackle such challenges of water quality [9]. These require a lot of research work that can provide an in-depth understanding of the current quality situation and of any factors influencing the groundwater chemistry [10], which is sadly lacking [11]; thus very little is known about the hydrochemistry status of groundwater in small tropical islands [5].

Multivariate statistical analyses such as principal component analysis (PCA) and cluster analysis (CA) provide a reliable alternative approach for understanding and interpreting the complex system of water quality with the capability of analyzing large amounts of data [9] and distinguishing complex relationships among many variables [12]. As such, multivariate statistical analysis has been used by many researchers all over the world to analyze water quality. For example, in India, Singh *et al.* [13] analyzed the suitability of groundwater in Imphal for drinking and for domestic and agricultural activities using CA, PCA, and factor analysis (FA). In northeastern Tunisia, Tlili-Zrelli *et al.* [14] used multivariate statistical analyses, combining PCA and hierarchical cluster analysis (HCA) to investigate natural and anthropogenic processes controlling the groundwater mineralization and quality. Multivariate statistical analysis was also used by Akbal *et al.* [9] to study water and sediment quality on the mid-Black sea coast of Turkey. By applying PCA, they were able to extract five and three factors that were responsible for 87.63% and 84.73% of the total variations in surface water and sediments, respectively. Shyu *et al.* [15] integrated factor analysis with kriging and information entropy theory to determine the stability of groundwater quality variation in Taiwan between 2005 and 2007.

This work puts multivariate statistical analysis to the test, with the aim of assessing the groundwater quality and evaluating various factors and processes influencing the hydrochemistry and quantitative contributions of each factor to the variations in groundwater chemistry of Kapas Island.

The choice of Kapas Island for this study is based on the fact that the island falls under the category of small islands [16] with a total area of about 2 km^2^ and there is no surface water available for use, therefore it relies totally on groundwater resources for domestic and other uses, leading to over-exploitation of the groundwater to meet the increasing demand. Furthermore, over 80% of the island is hilly, which means fewer recharge areas and runoff of most of the rainwater into the sea. Its geology and topography in addition to the sea bordering the island from all directions make Kapas Island vulnerable to pollution from human activities and seawater intrusion due to intense pumping of the groundwater, especially with the current increase in tourism. Therefore, to effectively and properly protect groundwater, it is crucial to be able to evaluate the factors influencing the groundwater chemistry and translate this information (which is currently lacking) into a reliable and sustainable water management strategy that will guide end-users, such as land and water-resources managers, to prevent or minimize harmful impacts on groundwater quality and to ensure sustainability of fresh water resources for both human use and ecosystems in general.

## 2. Study Area

**Figure 1:**
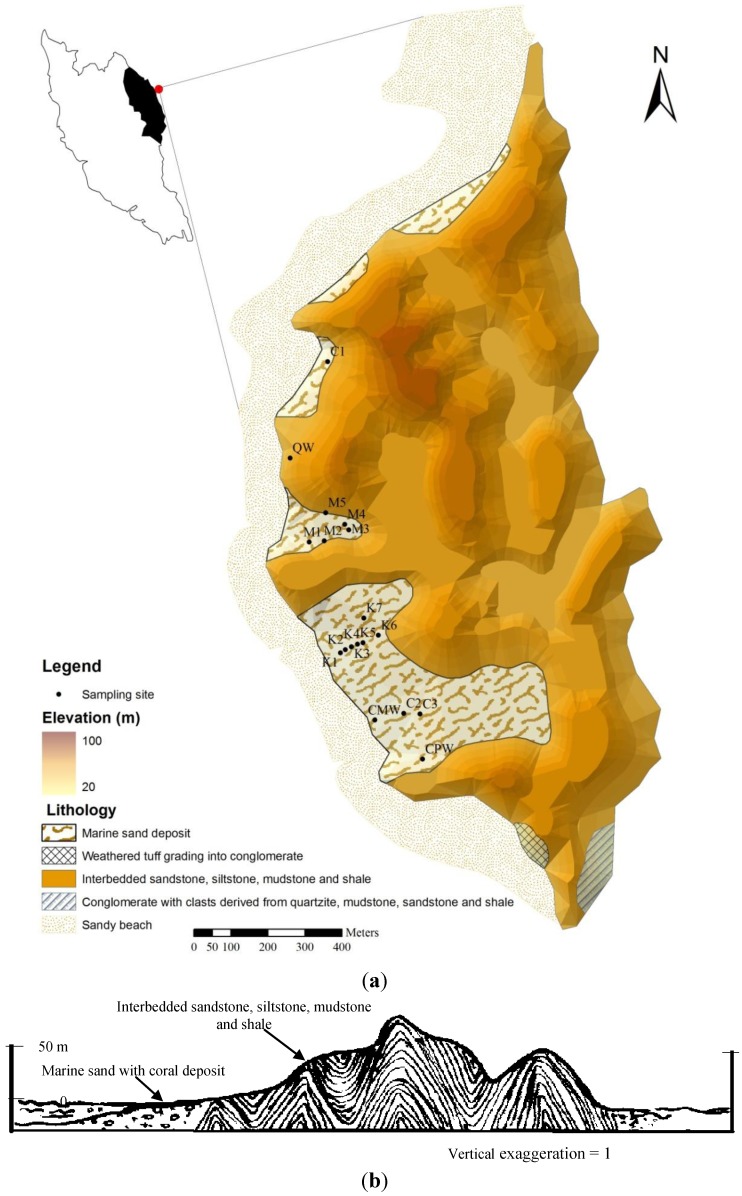
Map of study area showing sampling sites, elevation and lithology of the island (**a**) and (**b**) cross section of the island from west to east. Modified from [18].

Kapas Island is located in the northeastern part of Malaysia between 5°13.140'N and 103°15.894'E, as part of Terengganu State, bordering the South China Sea, and at a distance of about 3 km from the Marang coastal area. The total land area of the island is about 2 km^2^ [17]. More than 80% of the island is hilly, with a maximum elevation of 100 m. The island’s geology consists mostly of carbonaceous, interbedded sandstone, siltstone, mudstone and shale. While shaly siltstone, conglomerate with quartzite siltstone (Triassic-Jurassic age) and weathered tuff are found at the southern part of the island [18] (Figure 1). The climate of the island is characterized as tropical, receiving about 2,800 mm of rainfall annually, most of it during the monsoon season between November and February. The temperature fluctuates between 28 and 31 °C, with a humidity of about 80% [17].

## 3. Materials and Methods

### 3.1. Sampling and Chemical Analysis

For this work, 18 sampling sites (15 boreholes and 3 open wells) were utilized. Two sampling exercises were carried out for the months of August and September 2012, where 3 replicates of groundwater samples were collected during each sampling exercise from each of the 18 sampling sites (total of 108 samples for the 2 months) and were used to analyze the major ions. Prior to the sampling survey, the water containers (polyethylene bottles) underwent an acid wash with 5% nitric acid (HNO_3_) and were rinsed with distilled water. For every sampling point, the water level (W/L) was measured followed by emptying the tube-well three times or pumping the water out of the borehole for at least 10 min before taking water samples [17]. This was to ensure that the samples collected truly represented the groundwater and not the stagnant water that resides in the bore hole.

*In-situ* parameters such as dissolved oxygen (DO), pH, oxidation-reduction potential (*Eh*) electrical conductivity (EC), temperature (Temp) and turbidity (Tur) were measured in the field during sample collection. Major anions such as bicarbonate (HCO_3_) and chloride (Cl) were immediately analyzed by titration, while NO_3_ and SO_4_ were analyzed using a HACH (DR/2000) meter with 25 mL samples, as reported previously [19]. The samples were filtered using 0.45 μm filter paper (Whatman Milipores, Clifton, NJ, USA) and HNO_3_ acid was added to bring the pH below 2 [8,19]. The samples were then stored at 4 °C and transported to the laboratory for analyses of the major cations (Ca, Mg, Na and K) with an atomic absorption spectrophotometer (AAS) machine [17,20].

### 3.2. Statistical Analysis

Multivariate statistical analysis was applied for 17 water parameters utilizing correlation matrix, PCA and HCA to evaluate factors influencing the groundwater chemistry and sources of pollution in the island’s aquifer. First, the relationships between different water variables were determined using Pearson’s correlation coefficient.

### 3.3. Principal Component Analysis

PCA is the most widely used technique among the families of multivariate statistical analysis [21]. It is a technique which identifies patterns in data and then presents them based on their similarities and differences. Delineating patterns in data with a complex relation is not an easy task, thus utilizing PCA in such a case (for example, water chemistry) would provide a reliable result [22]. The main aim of PCA is to summarize a multivariate dataset by reducing the statistical noise in the data, exposing the outlier, and then arranging the components in descending order (from the largest contributor to the least) as accurately as possible with as few principal components as possible [23]. Normally the first few PCs will interpret the variables with the highest variance in the case of large differences in variance. Variables are normalized individually to unit variance and, as such, contribute equally when the correlation matrix is used [24]. In this study, a Pearson correlation matrix was used to determine the relationship between variables. The classification was based on Guildford’s rule of thumb for interpreting the Pearson product moment correlation [25] (Table 1).

**Table 1:** Guildford’s rule of thumb for interpreting correlation coefficient [25].

*r*-value	Interpretation
0.0 to 0.29	Negligible or little correlation
0.3 to 0.49	Low correlation
0.5 to 0.69	Moderate or marked correlation
0.7 to 0.89	High correlation
0.9 to 1.00	Very high correlation

The Kaiser-Mayer-Olkin (KMO) measure is applied first before executing the PCA; this is to evaluate the suitability of PCA and to test the adequacy of samples. It is advisable only to proceed to the next level if the KMO value is 0.5 and above [26]; in this study the KMO was found to be 0.646. PCA extracts variables into groups known as principal components (PCs), with their eigenvalues, variability (%) and cumulative values (%) of individual and collective PCs, and these are used to plot a graph from which the PCs with eigenvalue greater than 1 are retained.

### 3.4. Cluster Analysis

Cluster analysis is a multivariate statistical method that classifies variables into groups called clusters based on their similarity to each other and dissimilarity to the other groups. This classification is done objectively without prior assumptions regarding the data so as to uncover structure or underlying patterns of the original data set [27]. Here, cluster analysis was applied to the physical and chemical parameters of groundwater.

## 4. Results and Discussion

### 4.1. Statistical Summary

Table 2 presents the statistical summary of the groundwater hydrochemical parameters of Kapas Island for the months of August and September 2012. The groundwater pH shows both acidity and alkalinity, but the low pH 4.5 was found in the middle and the lowest parts of the island (Figure 1).

**Table 2:** Statistical summary of Kapas Island groundwater chemistry for the months of August–September 2012.

Variable	Units	Minimum	Maximum	Mean	Std. deviation
Ca	mg·L^−1^	1.1	254.4	69.9	48.2
Mg	mg·L^−1^	1.0	496.2	47.7	117.2
Na	mg·L^−1^	3.0	1,920.7	241.3	563.6
K	mg·L^−1^	0.5	83.3	14.0	24.2
HCO_3_	mg·L^−1^	48.8	7,320.0	613.1	1,511.0
Cl	mg·L^−1^	31.0	6,198.1	517.7	1,455.1
SO_4_	mg·L^−1^	1.0	500.0	70.3	134.6
NO_3_	mg·L^−1^	0.4	10.6	1.7	2.3
EC	µS·cm^−1^	138.6	14,085.0	1,756.5	3,463.4
Salinity	mg·L^−1^	0.1	7.9	0.9	1.9
pH	-	4.5	7.7	6.9	0.9
DO	mg·L^−1^	2.3	9.7	6.5	1.5
Tur	NTU	0.9	1,297.0	235.8	407.9
Temp	°C	27.4	30.5	28.8	1.0
W/L	m	1.1	3.3	2.4	0.5
Eh	mV	−20.8	195.4	25.9	58.1
TDS	mg·L^−1^	70.3	8,213.1	988.3	2,020.5

Moreover, this area was characterized by the lowest HCO_3_ 48 mg·L^−1^ and high SO_4_, which may be as a result of the runoff waterways that continue to moisturize the area and increase the dissolved organic carbon (DOC), eventually leading to a decrease in pH [28]. Furthermore, chemical reactions in the area may also influence redox reaction due to leaching from sewage; hence sewage and DOC are believed to have a significant influence on redox reaction [29]. Total dissolved solids (TDS) values on the island range from 70 to >8,000 mg·L^−1^, indicating the likelihood of seawater intrusion. TDS, EC and salinity were strongly correlated with all the major ions except NO_3_, which indicates that TDS, EC and salinity are influenced by these major ions. The order of abundance for the cations was found to be Na > Mg > Ca > K and for anions was HCO_3_> Cl > SO_4_ > NO_3_. The HCO_3_ was found to be the most abundant anion in the study area. The presence of HCO_3_ in the groundwater could be due to the dissolution of carbonate materials from the corals found in the marine sand deposits [18].

### 4.2. Correlation

**Figure 2:**
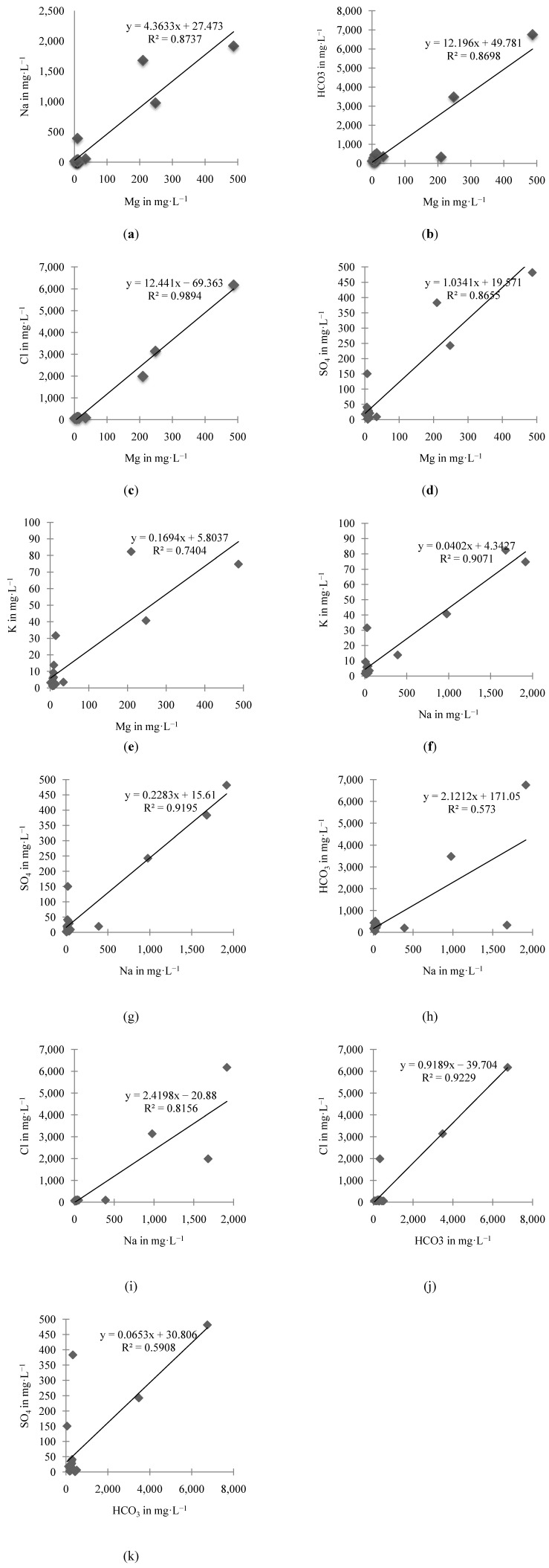
Bivariate linear graphs of the most significant parameters at *p* < 0.01.

The correlation matrix (Table 3) describes the interrelationship between variables [30], and the results for 17 hydrochemical parameters show that very high positive correlation exist between Mg-Na (*r* = 0.93, *p* < 0.01), Mg-HCO_3_ (*r* = 0.92, *p* < 0.01), HCO_3_-Cl (*r* = 0.95, *p* < 0.01), Mg-Cl (*r* = 0.99, *p* < 0.01), Mg-SO_4_ (*r* = 0.92, *p* < 0.01), Na-K (*r* = 0.95, *p* < 0.01), Na-Cl (*r* = 0.90, *p* < 0.01), Cl-SO_4_ (*r* = 0.90, *p* < 0.01), Na-SO_4_ (*r* = 0.95, *p* < 0.01) and K-SO_4_ (*r* = 0.90, *p* < 0.01) and very high negative correlation exists between pH and Eh (*r* = −0.96, *p* < 0.01). High positive correlation exist between Mg and K (*r* = 0.85, *p* < 0.01), Na and HCO_3_ (*r* = 0.73, *p* < 0.01) and HCO_3_-SO_4_ (*r* = 0.74, *p* < 0.01). There is high positive correlation between Ca and Mg (*r* = 0.7, *p* < 0.01), Ca-HCO_3_ (*r* = 0.74, *p* < 0.01), Ca-Cl (*r* = 0.71, *p* < 0.01) and K-Cl (*r* = 0.8, *p* < 0.01). A moderate positive correlation exists between K-HCO_3_ (*r* = 0.63, *p* < 0.01), K-Ca (*r* = 0.52, *p* < 0.05) and SO_4_-Ca (*r* = 0.55, *p* < 0.05), and a moderate negative correlation exists between DO-NO_3_ (*r* = –0.62, *p* < 0.01). EC, salinity and TDS have very high correlation of (*r* = 1, *p* < 0.01) with one another and very high, high and moderate correlations with Mg, Na, K, Cl, SO_4_, HCO_3_ and Ca (*r* = 0.99, 0.95, 0.91, 0.98, 0.94, 0.88 and 0.69 with *p* < 0.01), respectively. This suggests that EC, salinity and TDS are controlled by these ions [31]. Ca, Mg and HCO_3_ are the most abundant ions in natural water [32], while Na and Cl are the most abundant ions in seawater; therefore, a high correlation of Na and Cl in groundwater is an indication of saline water intrusion in coastal aquifers [33]. The relationships between the physicochemical parameters are given in Figure 2. From Table 3 it is quite clear that most of the parameters were significantly correlated with HCO_3_, which could be an indication that the aquifer system may have experienced various processes such as ion exchange, water-rock interaction and weathering of the aquifer’s parent material, therefore making HCO_3_ a dominant ion in the island water chemistry [17]. For example, the correlation matrix shows high positive correlation between Ca and HCO_3_, which indicates recharge [34]; at the same time it can also be an indication of weathering of calcite mineral, as illustrated in Equations (1) (carbonate acid formation) and (2) (calcite weathering equation):

H_2_O + CO_2_ → H_2_CO_3_ (1)

CaCO_3_ + CO_2_ + H_2_O → Ca^2+^ + 2HCO_3_^-^ (2)

First, H_2_O in the atmosphere reacts with CO_2_ to form carbonic acid (Equation (1)), then the rainwater falls on the land surface and dissolves part of the aquifer’s parent material, CaCO_3_ (calcite) [17], as shown in Equation (2). These processes give rise to surplus Ca and HCO_3_ ions, thus during the recharge process a lot of Ca and HCO_3_ ions are released into the groundwater. The correlation matrix also shows a significatant positive relation between HCO_3_, Mg, and Ca, which is an indication of weathering of dolomite, as shown in Equation (3):

CaMg(CO_3_)_2_ + 2CO_2_ + 2H_2_O → Ca^2+^ + Mg^2+^ + 4HCO_3_^-^ (3)

**Table 3:** Correlation matrix of the hydrochemical parameters.

Variables	Ca	Mg	Na	K	HCO_3_	Cl	SO_4_	NO_3_	EC	Salinity	pH	DO	Tur	Temp	W/L	Eh	TDS
Ca	**1**																
Mg	0.700 **	**1**															
Na	0.606 **	0.931 **	**1**														
K	0.524 *	0.853 **	0.949 **	**1**													
HCO_3_	0.737 **	0.920 **	0.731 **	0.632 *	**1**												
Cl	0.713 **	0.994 **	0.897 **	0.809 **	0.950 **	**1**											
SO_4_	0.550 *	0.924 **	0.954 **	0.904 **	0.743 **	0.899 **	**1**										
NO_3_	−0.056	0.101	0.164	**0.440**	0.036	0.069	0.127	**1**									
EC	0.688 **	0.990 **	0.955 **	0.913 **	0.878 **	0.976 **	0.941 **	0.206	**1**								
Salinity	0.689 **	0.992 **	0.954 **	0.909 **	0.881 **	0.978 **	0.942 **	0.195	1.000 **	**1**							
pH	0.246	−0.109	−0.058	−0.037	−0.134	−0.142	−0.228	0.029	−0.083	−0.091	**1**						
DO	0.052	0.042	0.039	−0.142	0.071	0.069	0.062	−0.622 **	−0.019	−0.007	**−0.391**	**1**					
Tur	−0.086	−0.200	−0.217	−0.252	−0.151	−0.181	−0.187	−0.114	−0.211	−0.216	**−0.287**	0.086	**1**				
Temp	−0.152	−0.224	**−0.323**	**−0.305**	−0.126	−0.190	**−0.286**	0.058	−0.225	−0.226	0.057	**−0.379**	0.048	**1**			
W/L	0.128	−0.055	−0.110	−0.114	0.050	−0.025	−0.185	0.031	−0.063	−0.072	**0.308**	−0.493 *	0.249	**0.351**	**1**		
Eh	**−0.328**	0.039	−0.024	−0.047	0.078	0.071	0.184	−0.075	0.005	0.012	−0.964 **	**0.329**	**0.287**	−0.073	−0.235	**1**	
TDS	0.689 **	0.991 **	0.953 **	0.910 **	0.882 **	0.977 **	0.940 **	0.202	1.000 **	1.000 **	−0.088	−0.015	−0.210	−0.223	−0.063	0.010	**1**

** Correlation is significant at the 0.01 level (2-tailed). * Correlation is significant at the 0.05 level (2-tailed).

A very high positive correlation exists between HCO_3_ and Na, Cl and Ca; this is an indication of interaction between fresh and saltwater. The concentrations of Ca in the groundwater may decrease while the concentration of Na increases during groundwater mixing, which are the interactions with the aquifer matrix. The Ca ions are substituted by the Na ions on the solid surface, as demonstrated in the equation below:

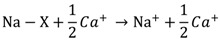
(4)

X is considered to be a soil exchanger, and the groundwater comes into contact with Na, which originated from seawater or halite minerals (NaCl) that precipitated on the aquifer matrix. The Na ion pushes away the Ca ion and thereby becomes dominant in the groundwater through the process of cation exchange. As such, Na ion increases in the groundwater solution [17], while Cl remains unchanged due to its ability to resist changes.

A high correlation between HCO_3_ and SO_4_ could mean intensive weathering [35], but detection of sulfate concentrations in an aquifer formed of carbonate rocks is rather complicated, as it means that there is a significant amount sulfur minerals in the carbonate aquifers. This requires an alternative explanation because carbonate aquifers hardly contain measurable concentrations of sulfur minerals [36]. However, during the groundwater sampling survey, sulfide odors in the groundwater samples were noted, suggesting the presence of hydrogen sulfide in the groundwater of the island. This justifies an earlier finding [17] which suggested that a sulfate reduction process is responsible for the odor, as shown in the following equation:

SO_4_^2-^ + 2HC_2_ O ↔ H_2_S + 2HCO_3_^-^ (5)

An anaerobic environment such as a closed groundwater system on an island is suitable for the growth of sulfate-reducing bacteria (SRB) and, subsequently, the development of H_2_S in groundwater. Some part of the island is undergoing reclamation activities, which might be the reason for the increase in H_2_S concentrations. Therefore, the main source for SO_4_ is probably due to H_2_S reduction, as hydrochemical conditions within the aquifer are generally reduced [17]. The enrichment in SO_4_ can also originate from degradation of organic substances from topsoil and runoff water, leached sulfates from agricultural areas due to fertilizer application, sewage and other human activities [36]. The moderate negative correlation between DO and NO_3_ is an indication of anthropogenic pollution with lots of microbial activity in the area [31]. The high negative correlation between Eh and pH is a sign of the stability of water and shows that most of the redox reactions on the island are pH dependent [37].

### 4.3. Extraction of Components

**Figure 3:**
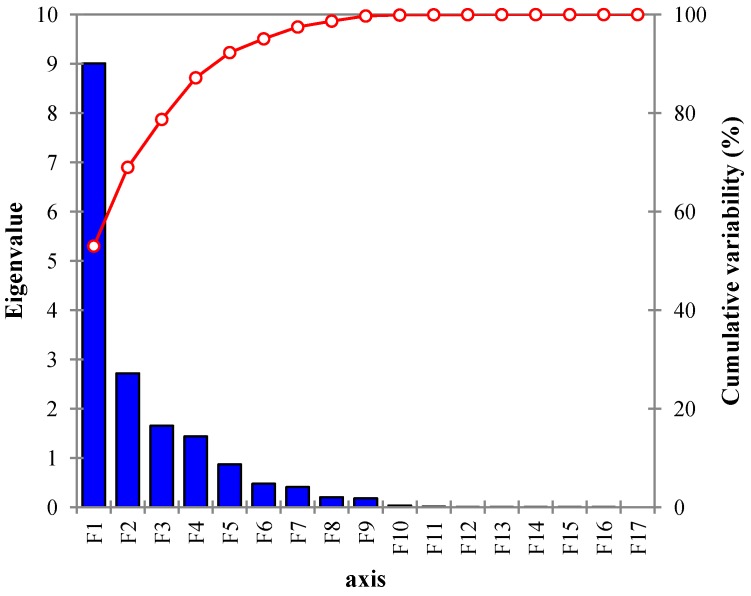
Scree plot of principal components.

PCA was applied to the 17 physiochemical parameters and it yielded 17 PCs (Table 4), but only PCs with eigenvalue greater than 1 are considered to be the most important [1,38], thus four PCs appear to be significant (Figure 3, Table 5), and the higher the eigenvalue of a PC, the greater the contribution of that particular PC to the variability of the groundwater chemistry [39]. For the interpretation of the factors that are of high significance without changing the variance, factor rotation using varimax, which is the most popular rotation technique [40], was employed.

**Table 4:** Eigenvalue, variability and cumulative % of each of the extracted components.

Components	Eigenvalue	Variability (%)	Cumulative %
**PC1**	**9.008**	**52.986**	**52.986**
**PC2**	**2.725**	**16.028**	**69.015**
**PC3**	**1.654**	**9.732**	**78.747**
**PC4**	**1.418**	**8.340**	**87.087**
PC5	0.872	5.129	92.216
PC6	0.474	2.786	95.002
PC7	0.407	2.394	97.396
PC8	0.215	1.266	98.662
PC9	0.175	1.027	99.690
PC10	0.035	0.206	99.896
PC11	0.009	0.054	99.950
PC12	0.005	0.027	99.977
PC13	0.002	0.014	99.990
PC14	0.001	0.007	99.997
PC15	0.000	0.003	100.000
PC16	0.000	0.000	100.000
PC17	0.000	0.000	100.000

Components in bold are considered to be the most significant.

According to Kaiser’s criterion, otherwise known as the eigenvalue-one criterion, which is one of the most commonly used criteria for solving the number-of-components problem in PCA [40], one should only retain and interpret any component with an eigenvalue greater than 1.00. This is because each of the observed variables contributes one unit of variance to the total variation in the data set. As such, any component that displays an eigenvalue greater than 1.00 is believed to be responsible for a greater amount of variation than is contributed by one variable. Thus a component with such a characteristic is responsible for a significant amount of variance and deserves to be retained, whereas a component with an eigenvalue less than 1.00 is responsible for less variation than is contributed by one variable. However, the main aim of PCA is to decrease the number of observed variables to a relatively smaller number of components without jeopardizing the actual interpretation of the data in question; therefore retaining components that account for less variance than contributed by individual variables defeats the aim of PCA. For this reason, components with eigenvalues less than 1.00 are viewed as trivial and are not retained [41].

**Table 5:** Factor loadings after varimax rotation.

Variables	PC1	PC2	PC3	PC4
Ca	**0.752**	−0.362	−0.280	0.135
Mg	**0.991**	0.017	−0.004	−0.070
Na	**0.934**	−0.027	0.097	−0.211
K	**0.865**	−0.033	0.364	−0.232
HCO_3_	**0.912**	0.036	−0.130	0.093
Cl	**0.987**	0.045	−0.049	−0.023
SO_4_	**0.922**	0.154	0.091	−0.215
NO_3_	0.123	0.007	**0.908**	0.001
EC	**0.989**	−0.004	0.100	−0.091
Sal	**0.989**	0.001	0.089	−0.098
pH	−0.088	**−0.968**	0.054	0.112
DO	0.012	0.299	**−0.762**	−0.467
Tur	−0.147	**0.444**	−0.246	**0.503**
Temp	−0.205	0.000	0.197	**0.635**
W/L	0.019	−0.226	0.053	**0.848**
Eh	0.016	**0.966**	−0.053	−0.074
TDS	**0.990**	0.000	0.094	−0.089
Eigenvalue	9.009	2.719	1.653	1.436
Variability (%)	52.993	15.995	9.726	8.450
Cumulative %	52.993	68.988	78.714	87.164

The PCA result consists of four PCs that cumulatively account for 87% of the total variance in the hydrochemistry. The first component, PC1, which normally accounts for the most significant process [42], explains 52.99% of the total variance with an eigenvalue of 9. The PC consists of all the major ions with the exception of NO_3_. These ions show strong positive loading with salinity, TDS and EC, suggesting that TDS, salinity and EC depend on contributions from the major ions. This component indicates an interaction between seawater and fresh water. It can also be noticed that Ca is relatively low compared to other major ions, especially Na; this is due to a cation exchange process, which takes place naturally when seawater intrudes in an aquifer [3]. One of the major advantages of PCA in the hydrochemical analysis is its ability to interpret each factor based on specific or multiple hydrochemical processes [43]. It is possible to have a single PC with multiple processes, as reported previously [1,42,43,44]. This is also the case for PC1(Table 5), where apart from the chemical process of seawater intrusion, other processes such as high loading of Ca-HCO_3_, which results from recharge or fresh water [5], and strong loading of HCO_3_-Mg and Ca indicate weathering [35], even though seawater intrusion is the dominant process.

PC2 accounts for 15.9% of the variance, which consists of a strong positive loading of Eh and strong negative loading of pH with a weak loading of Tur. As explained by Kehew [45], understanding of contaminants’ hydrogeology is impossible without the knowledge of the redox process taking place in a given aquifer. This is because Eh is directly or indirectly responsible for biochemical reactions and the activities of microorganisms, especially with regards to the process of biodegradation involving oxidation or reduction as well as the mobility of many elements. Hence Eh changes with the increase in organic matter [46], boosting activity by microorganisms and eventually creating a reduction in oxygen due to high oxygen demand. On the other hand, pH shows negative loading, which is likely influenced by the increase in DOC from runoff and leached from sewage, making the Eh reaction in the area dependent on the pH. A similar relationship between redox and pH was also reported by Morales *et al.* [47]. PC2 indicates a weak loading of turbidity, which can be explained by the fact that turbid water contains organic matter that would eventually attract microorganisms. 

The third PC has 1.6 eigenvalue, explains 9.7% of the total variability and consists of strong positive loading of NO_3_ and strong negative loading of DO. This suggests anthropogenic pollution [31]. This can be explained by the fact that NO_3_, which is an indicator of human pollution (usually sourced from domestic activities, sewage, agriculture and DOC), attracts microorganisms, thereby elevating the oxygen demand and leading to the depletion of DO.

PC4 accounts for 8.4% of the total variability with 1.4 eigenvalue. It consists of strong loading of water level (W/L) and moderate loading of Temp and Tur. This PC represents the natural or weather factor [28] where the strong loading in W/L shows recharge by rainfall creating more Tur and together altering the Temp.

### 4.4. Cluster Analysis

Cluster analysis was applied to the groundwater variables and the resulting factors were classified into three major groups based on the similarities among the variables and dissimilarities to other groups.

**Figure 4:**
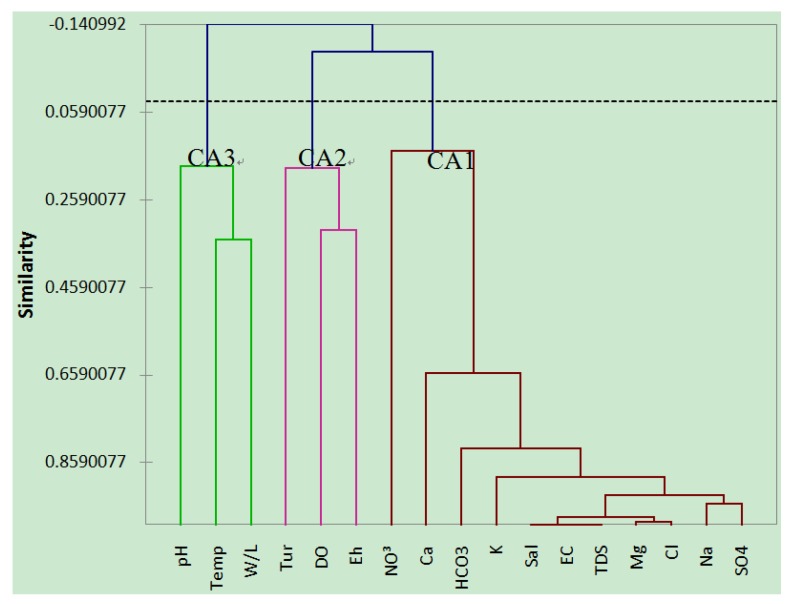
Dendrogram based on the clustering of groundwater quality data.

The first group, CA1, as shown in Figure 4, consists of all the major ions that strongly influenced TDS, EC and Sal. This group explains multiple processes affecting the groundwater, ranging from NO_3_, which is indicative of anthropogenic pollution [31], to Ca-HCO_3_, HCO_3_-SO_4_, and Mg-Na-Cl, which are indicative of freshwater recharge [34], weathering or leached from sewage [35] and seawater intrusion [3], respectively.

The second group, CA2, contains Tur, DO and Eh. Microbial activity and organic matter are associated with an increased turbidity [48]. In line with this, microbial activity causes an increase in oxygen demand, which affects DO. As the DO decreases, Eh is altered giving rise to change in hydrogen ions in water. As explained by Nelson [28], Eh, Temp and pH are responsible for chemical reactions taking place in groundwater.

The last group, CA3, consists of three variables, namely pH, Temp and W/L. This group can be categorized as weather or natural factor because virtually all the group members are directly or indirectly associated with weather. Usually, rainwater is slightly acidic [28] and this acidity increases as the runoff carries organic materials and recharges the aquifer. When the water level rises and the temperature is altered, higher temperature increases chemical and microbial activities. This is because temperature is a major factor affecting almost all physiochemical equilibriums and biological reactions in water [49].

Note that there is a little variation or adjustment, as the case may be, between PCA grouping and CA classification, as it can be seen that PCA grouped the variables into four groups of factors, namely seawater intrusion, redox reaction, anthropogenic and natural factors (see Table 4), while CA classified the variables into three classes: seawater intrusion, redox reaction and natural factors (Figure 3). This might have to do with the fact that PCA is a statistical tool that extracts components from a random matrix of original variables while CA regroups variables by classifying them into their true groups based on similarities to one another and their dissimilarities to other group members [1]. For example, the first component here, which is PCA1, consists of all the major ions with the exception of NO_3_, and these major ions are believed to be responsible for the salinity, EC and TDS of the water [31]. However, in CA, the first CA class (CA1), which reflects the same factors as PCA1, has an additional member, NO_3_, which was originally a member of PCA3. This is likely due to the fact that NO_3_ concentration in the island’s groundwater is relatively uniform and low, approximately 1 mg·L^−1^, except for one sampling site, K5, where it was recorded as 10 mg·L^−1^. This is clearly an isolated case, as shown by other sampling sites. The reason for high concentration of NO_3_ in this particular sampling site may have to do with its proximity to a septic tank, which was located almost 30 m away from that site, and leaching from sewage is known to be one of the major sources of NO_3_ pollution in groundwater [29,31]. Another interesting fact is how PCA and CA demonstrate the interrelationship between grouping by PCA and the classification by CA. For example, CA3 reflects PCA4 with little alteration with regards to regrouping the variables. In this CA, Tur is replaced by pH, which can be attributed to rainwater, which is slightly acidic represented by pH, triggers the Tur through runoff, as the water carries a variety of materials, including organic matter, and then recharges the aquifer which raises the W/L and alters the temperature. Increased temperature provides organisms with a more suitable environment for their activities, which in turn affects Eh. This explains how all the variables in this group (Temp, W/L and pH) are interrelated.

The grouping of variables by CA justifies the earlier classification by PCA with few adjustments. As can be recalled, the grouping of variables was based on similarities and dissimilarities among variables with respect to processes affecting them [27].

### 4.5. Groundwater Classification

**Figure 5:**
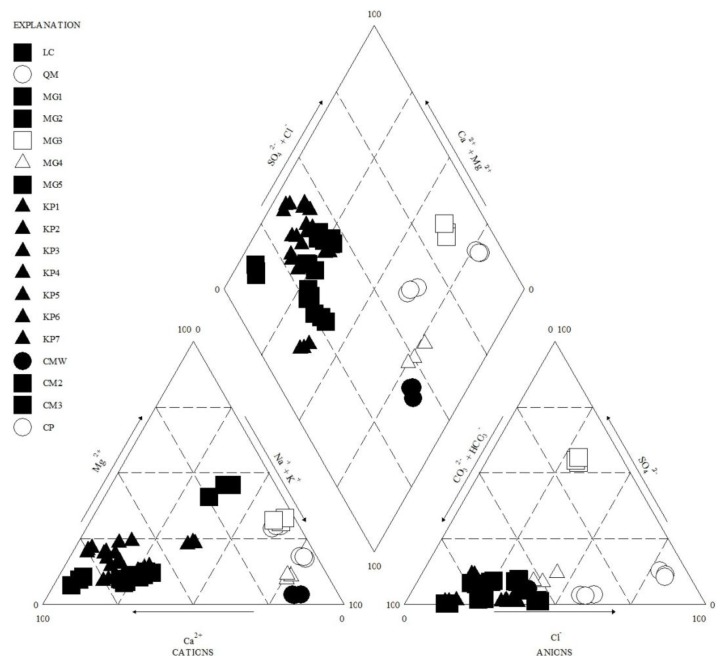
Piper plot of the groundwater samples of Kapas Island (for the months of August–September 2012).

The classification of groundwater types was based on a trilinear Piper diagram [50], where all major ions are used for the classification (Figure 5). Most of the samples are within the Ca-HCO_3_ water type, which can be interpreted as fresh or recharge water [5,34]. The second water type represented by M4 and CMW (Figure 1) sampling sites on the island is Na-HCO_3_ water type, suggesting shifting of water type from Ca-HCO_3_ to Na-HCO_3_ [17] or water-rock interactions [35]. Na-Cl water type was found in QW and CPW sampling sites; both are open wells, characterized by high EC, TDS and salinity, and have been in use for over a decade; moreover, these sampling sites are located less than 50 m from the sea. This suggests that the continuous exploitation of groundwater has led to seawater intrusion [5].

The last water type on the island, represented by M3 sampling point, was found to be Na-SO_4_-Cl type, yet it was not classified as seawater intrusion because this chemical composition is not similar to that of seawater intrusion in the island. This sampling site has the lowest pH (4.5) and HCO_3_ in the area and, at the same time, contains high SO_4_ concentrations. The occurrence of high SO_4_ concentrations in parts of the island (e.g., M3) is believed to be from either of the following sources or a combination of both. Firstly, the fact that the sampling exercise took place in pre-monsoon season, this show that high value of SO_4_ in some parts of the island groundwater is likely source from the breaking of organic substances from topsoil/water [36]. Secondly, the island is highly congested with many sewage systems, septic-tank and other sanitary disposal systems, which significantly contributes SO_4_ into the shallow aquifer. Thus, the low concentration of NO_3_ or SO_4_ in most parts of the island doesn’t mean that the groundwater in those areas has not been contaminated by leached from septic tanks, and other methods of sanitary disposal, but rather, the redox reactions in the island aquifer as indicated in Equation (5) hurriedly reduces both SO_4_ and NO_3_ from the groundwater [51].

## 5. Conclusions

Physical and chemical parameters of Kapas Island were successfully analyzed with PCA and HCA to evaluate factors influencing the groundwater chemistry of the area. The hydrochemistry of the island’s groundwater was found to be influenced by four factors, namely seawater intrusion, redox reaction, anthropogenic pollution and weather factor. PCA results suggest that the first component is the highest and the most influential factor regarding the chemistry of the groundwater, and it involves multiple processes (recharge, weathering and seawater intrusion), the dominant of which is seawater intrusion. Except for NO_3_ that was characterized as the PC3 source from anthropogenic pollution, all major ions contributed in PC1 and together they controlled TDS, EC and salinity. PC2 and PC4 were found to be influenced by redox reactions and natural or weather factors, respectively. The resulting output of HCA justified the earlier interpretation of the influencing factors by PCA.

Furthermore, the Piper diagram suggests that most of the sampling points fall within the Ca-HCO_3_ fresh water type, while CW and M4 sampling sites were found to be Na-HCO_3_ mixed water types. M3 sampling site, on the other hand, had Na-SO_4_-Cl water type, indicating redox reaction and leaching from sewage. Additionally, QW and CPW sampling sites were identified as Na-Cl water types, indicating seawater intrusion, which was not surprising as both wells were situated near the sea and have been in use for more than 10 years.

## References

[1] Arslan H. (2013). Application of multivariate statistical techniques in the assessment of groundwater quality in seawater intrusion area in Bafra Plain, Turkey. Environ. Monit. Assess..

[2] Trabelsi R., Zairi M., Dhia H.B. (2007). Groundwater salinization of the Sfax superficial aquifer, Tunisia. Hydrogeol. J..

[3] Aris A.Z., Abdullah M.H., Ahmed A., Woong K.K. (2007). Controlling factors of groundwater hydrochemistry in a small Island’s aquifer. Int. J. Environ. Sci. Tech..

[4] Praveena S.M., Abdullah M.H., Bidin K., Aris A.Z. (2011). Understanding of groundwater salinity using statistical modeling in a small tropical island, East Malaysia. Environmentalist.

[5] Aris A.Z., Abdullah M.H., Woong K.K., Praveena S.M. (2009). Hydrochemical changes in small tropical island’s aquifer: Manukan Island, Sabah, Malaysia. Environ. Geol..

[6] Mohapatra P., Vijay R., Pujari P., Sundaray S., Mohanty B. (2011). Determination of processes affecting groundwater quality in the coastal aquifer beneath Puri city, India: A multivariate statistical approach. Water Sci. Tech..

[7] Singh C.K., Shashtri S., Mukherjee S. (2011). Integrating multivariate statistical analysis with GIS for geochemical assessment of groundwater quality in Shiwaliks of Punjab, India. Environ. Earth Sci..

[8] Belkhiria L., Mounib L., Boudoukha A. (2012). Geochemical evolution of groundwater in an alluvial aquifer: Case of El Eulma aquifer, East Algeria. J. Afr. Earth Sci..

[9] Akbal F., Gürel L., Bahadır T., Güler İ., Bakan G., Büyükgüngör H. (2011). Water and sediment quality assessment in the mid-Black Sea coast of Turkey using multivariate statistical techniques. Environ. Earth Sci..

[10] Glynn P.D., Plummer L.N. (2005). Geochemistry and the understanding of ground-water systems. Hydrogeol. J..

[11] Aris A.Z., Abdullah M.H., Woong K.K. (2007). Hydrogeochemistry of groundwater in Manukan Island, Sabah. Malays. J. Anal. Sci..

[12] Jalali M. (2010). Application of Multivariate analysis to study water chemistry of groundwater in a semi-arid aquifer, Malayer, Western Iran. Desalination Water Treat..

[13] Singh E.J.K., Gupta A., Singh N.R. (2012). Groundwater quality in Imphal West district, Manipur, India, with multivariate statistical analysis of data. Environ. Sci. Pollut. Res..

[14] Tlili-Zrelli B., Hamzaoui-Azaza F., Gueddari M., Bouhlila R. (2012). Geochemistry and quality assessment of groundwater using graphical and multivariate statistical methods. A case study: Grombalia phreatic aquifer (Northeastern Tunisia). Arab. J. Geosci..

[15] Shyu G.-S., Cheng B.-Y., Chiang C.-T., Yao P.-H., Chang T.-K. (2011). Applying factor analysis combined with kriging and information entropy theory for mapping and evaluating the stability of groundwater quality variation in Taiwan. Int. J. Environ. Res. Public Health.

[16] White I., Falkland T., Perez P., Dray A., Metutera T., Metai E., Overmars M. (2007). Challenges in freshwater management in low coral atolls. J. Cleaner Prod..

[17] Isa N.M., Aris A.Z., Sulaiman W.N.A. (2012). Extent and severity of groundwater contamination based on hydrochemistry mechanism of sandy tropical coastal aquifer. Sci. Total Environ..

[18] Abdullah M. (1981). Geological Survey Report of Kapas Island.

[19] Association A.W.W., Federation W.E. (2005). Standard Methods for the Examination of Water and Wastewater.

[20] Parizi H.S., Samani N. (2012). Geochemical evolution and quality assessment of water resources in the Sarcheshmeh copper mine area (Iran) using multivariate statistical techniques. Environ. Earth Sci..

[21] Ranjan R.K., Ramanathan A., Parthasarathy P., Kumar A. (2012). Hydrochemical charasteristics of groundwater in the plains of Phalgu River in Gaya, Bihar, India. Arab. J. Geosci..

[22] Smith L.I. A Tutorial on Principal Component Analysis. http://csnet.otago.ac.nz/cosc453/student_tutorials/principal_components.pdf.

[23] Pitkänen P., Partamies S., Luukkonen A. (2004). Hydrogeochemical Interpretation of Baseline Groundwater Conditions at the Olkiluoto Site.

[24] Farnhama I., Johannessonb V., Singhc A., Hodged V., Stetzenbach K. (2003). Factor analytical approaches for evaluating groundwater trace element chemistry data. Anal. Chim. Acta.

[25] Guildford J.P. (1973). Fundamental Statistics in Psychology and Education.

[26] Mustapha A., Aris A.Z. (2012). Multivariate statistical analysis and environmental modeling of heavy metals pollution by industies. Pol. J. Environ. Stud..

[27] Mohapatra P., Vijay R., Pujari P., Sundaray S., Mohanty B. (2011). Determination of processes affecting groundwater quality in the coastal aquifer beneath Puri city, India: A multivariate statistical approach. Water Sci. Tech..

[28] Nelson D. (2002). Natural Variations in the Composition of Groundwater.

[29] Abrahams R.H., Loague K. (2008). A compartmentalized solute transport model for redox zones in contaminated aquifers: 2. Field-scale simulations. Water Resour. Res..

[30] Mor S., Singh S., Yadav P., Rani V., Rani P., Sheoran M., Singh G., Ravindra K. (2009). Appraisal of salinity and fluride in a semi-arid region of India using statistical and multivariate techniques. Environ. Geochem. Health.

[31] Sundaray S.K. (2010). Application of multivariate statistical techniques in hydrogeological studies—A case study of Brahmani-Koel River (India). Environ. Monit. Assess..

[32] Wollast R., Mackenzie F. (1983). Global Cycle of Silica. Silicon Geochemistry and Biogeochemistry.

[33] Panteleit B., Kessels W., Kantor W., Schulz H. Geochemical Characteristics of Salinization-Zones in the Coastal Aquifer Test Field (CAT-Field) in North-Germany. Proceeding of 5th International Conference on Saltwater Intrusion and Coastal Aquifers-Monitoring, Modeling, and Management.

[34] Thilagavathi R., Chidambaram S., Prasanna M.V., Thivya C., Singaraja C. (2012). A study of groundwater geochemistry in layered aquifers system of Pondicherry region, southeast India. Appl. Water Sci..

[35] Chidambaram S., Anandhan P., Prasanna M.V., Srinivasamoorthy K., Vasanthavigar M. (2012). Major ion chemistry and identification of hydrogeochemical processes controlling groundwater in and around Neyveli Lignite Mines, Tamil Nadu, South India. Arab. J. Geosci..

[36] Srinivasamoorthy K., Chidambaram S., Prasanna M.V., Vasanthavihar M., Peter J., Anandhan P. (2008). Identification of major sources controlling groundwater chemistry from a hard rock terrain—A case study from Mettur taluk, Salem district, Tamil Nadu, India. J. Earth Syst. Sci..

[37] Deutsch W.J. (1997). Groundwater Geochemistry: Fundamentals and Applications to Contamination.

[38] Pathak H., Limaye S.N. (2011). Study of seasonal variation in groundwater quality of sagar city (India) by principal component analysis. J. Chem..

[39] Abdi H. (2003). Factor Rotations in Factor Analyses. Encyclopedia of Social Science Research Methods.

[40] Kaiser H.F. (1960). The application of electronic computers to factor analysis. Educ. Psychol. Meas..

[41] O’Rourke N., Hatcher L., Stepanski E.J. (2005). A Step-by-Step Approach to Using SAS for Univariate & Multivariate Statistics.

[42] Yidana S.M., Yakubo B.B., Akabzaa T.M. (2010). Analysis of groundwater quality using multivariate and spatial analyses in the Keta basin, Ghana. J. Afr. Earth Sci..

[43] Suk H., Lee K.-K. (1999). Characterization of a water hydrochemical system through multivariate analysis: Clustering into groundwater zones. Groudwater.

[44] Kumaresan P.R.M. (2008). Factor analysis and linear regression model (LRM) of metal speciation and physico-chemical characters of groundwater samples. Environ. Monit. Assess..

[45] Kehew E.A. (2000). Applied Chemical Hydrogeology.

[46] Dia A., Gruau G., Olivié-Lauquet G., Riou C., Molénat J., Curmi P. (2000). The distribution of rare earth elements in groundwaters: Assessing the role of source-rock composition, redox changes and colloidal particles. Geochim. Cosmochim. Ac..

[47] Morales L.A., Paz-Ferreiro J., Vieira S. R., Vázquez E.V. (2010). Spatial and temporal variability of Eh and pH over a rice field as related to lime addition. Bragantia.

[48] Mann A.G., Tam C.C., Craig H.D., Rodrigues L.C. (2007). The association between drinking water turbidity and gastrointestinal illness: A systematic review. BMC Public Health.

[49] Delpla I., Jung A.-V., Baures E., Clement M., Thomas O. (2009). Impacts of climate change on surface water quality in relation to drinking water production. Environ. Int..

[50] Piper A.M. (1953). A Graphic Procedure in the Geochemical Interpretation of Water Analysis.

[51] McArthur J., Sikdar P., Hoque M., Ghosal U. (2012). Waste-water impacts on groundwater: Cl/Br ratios and implications for arsenic pollution of groundwater in the Bengal Basin and Red River Basin, Vietnam. Sci. Total Environ..

